# Glycopatterns and Glycoproteins Changes in MCN and SCN: A Prospective Cohort Study

**DOI:** 10.1155/2019/2871289

**Published:** 2019-07-31

**Authors:** Ying Wang, Yufa Sun, Jia Feng, Zheng Li, Hanjie Yu, Xiang Ding, Fuquan Yang, Enqiang Linghu

**Affiliations:** ^1^Department of Gastroenterology, The Affiliated Fu Xing Hospital of Capital Medical University, Beijing 100038, China; ^2^Department of Health Care, Central Guard Bureau, Beijing 100034, China; ^3^Department of Gastroenterology, Bethune International Peace Hospital, Shi Jia Zhuang 050082, China; ^4^Laboratory for Functional Glycomics, College of Life Sciences, Northwest University, Xi'an 710069, China; ^5^Laboratory of Proteomics, Institute of Biophysics, Chinese Academy of Sciences, Beijing 100101, China; ^6^Department of Gastroenterology, Chinese PLA General Hospital, Beijing 100853, China

## Abstract

*Background. *Advances in imaging improve the detection of malignant pancreatic cystic including mucinous cystic neoplasm (MCN), intraductal papillary mucinous neoplasm (IPMN), and mucinous cystic adenocarcinoma (MCA), but the distinction between benign and malignant lesions remains a problem. In an effort to establish glycopatterns as potential biomarkers for differential diagnosis between MCN and SCN, we systematically investigated the alterations of glycopatterns in cystic fluids for both SCN and MCN.* Methods. *Among the 75 patients enrolled, 37 were diagnosed as MCN and 38 as SCN based on histology. Lectin microarray analysis was performed on each sample, and the fluorescence intensity was used to obtain the fold-change. Then, mixed cyst fluids of MCN group and SCN group were cross bonded with magnetic particles coupled by Lectin STL and WGA, respectively. Hydrophilic interaction liquid chromatography (HILIC) enrichment was performed, liquid chromatography (LC)/mass spectrometry (MS) analysis and bioinformatical analysis was conducted to find the differential glycoproteins between MCNs and SCNs.* Results*. Through analysis of lectin microarray between MCNs and SCNs, stronger lectin signal patterns were assigned to Lectin WFA, DBA, STL, WGA, and BPL; and weaker signal patterns were assigned to Lectin PTL-I, Con A, ACA, and MAL-I. The glycoproteins were enriched by STL or WGA-coupled magnetic particles. Furthermore, the 10 identified correspondding genes were found to be significantly elevated in the mucinous cystadenoma: CLU, A2M, FGA, FGB, FGG, PLG, SERPINA1, SERPING1, C5, C8A, and C9. Bioinformatics analysis revealed that the above genes may activate the KEGG pathway: immune complement system.* Conclusion*. This study shows changes in glycopatterns and glycoproteins are associated with MCNs and SCNs.

## 1. Introduction

Based on imaging studies, pancreatic cystic neoplasms (PCNs) are being diagnosed with increasing frequency, the prevalence of PCNs in the general population is estimated to be between 2.6% and 45% [1–4]. Clinical differential diagnosis between the potential malignant pancreatic mucinous cystic neoplasm (MCN) and the mostly benign serous cystic neoplasm (SCN) remains challenging [5–8]. According to the Cochrane evidence-based guideline on PCNs, there are no clinical biomarkers of DNA, RNA, or protein in the blood to differentiate PCNs, high-grade dysplasia, or adenocarcinoma [9–12]. According to the evidence, carcinoembryonic antigen (CEA) >192ng/L in cyst fluids could be reports on potential malignant PCNs. Mostly, gene mutations of KRAS at codon 12,13 are hallmark genetic alterations of pancreatic ductal adenocarcinoma (PDAC), and GNAS codon 201 mutations are hallmark genetic alterations of IPMNs [13, 14].

Back in 2004, Hägglund first applied hydrophilic interaction liquid chromatography (HILIC) enrichment method to glycopeptide separation [15]. Nowadays, HILIC is a separation and analysis method based on positive phase chromatography with water/organic phase as mobile phase and polar material as fixed phase [16, 17]. With its developments over years, HILIC has become an important method mainly used to enrich glycosylated peptides.

**Figure 1:**
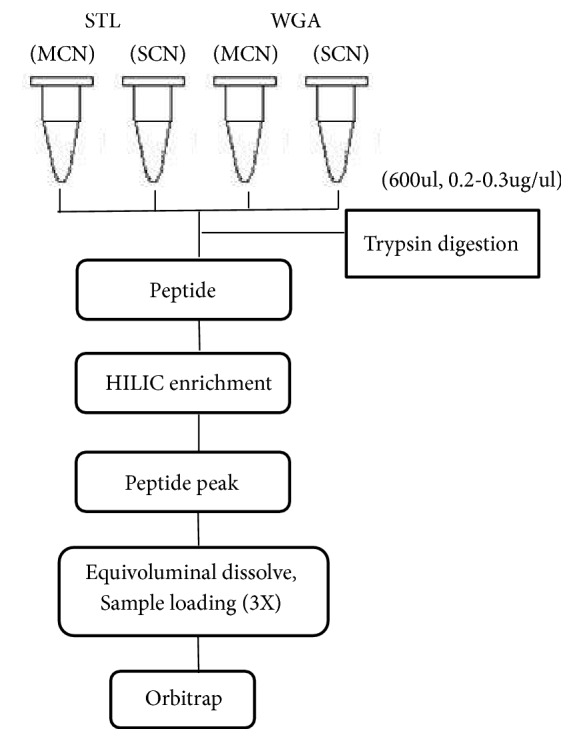
The flow chart of differential lectins enriched the glycoproteins.

Aberrant protein glycosylation often occurs during malignant transformation and leads to the expression of specific tumour-associated glycans [18–20]. Therefore, changes in glycosylation of proteins have the tremendous potential to be used as biomarkers to help the diagnosis of malignant diseases [21–23]. We report 75 cases, with 37 in the MCN group and 38 in the SCN group, using lectin microarrays to quantitatively analyse the differential types of glycan patterns and using the magnetic particles of labelled lectins to enrich the glycoproteins. Then, trypsin was used to digest proteins to peptides, and HILIC enrichment was performed. Equal sample volumes were dissolved and loaded for 3 times, and then liquid chromatography (LC) and mass spectrometry (MS) were used to identify the protein. The aim of this paper was to study the differential expression profiles of glycan-related proteins in MCNs using lectin microarrays. The overall strategy is summarized in Figure 1.

## 2. Materials and Methods

### 2.1. Study Design

This prospective study was approved by Fu Xing Hospital, Capital Medical University ethics committee (Approval Notice no. 2017FXHEC—KY044). The clinical records, EUS images, pathology, and surgical reports in this study were confirmed to be accurate.

### 2.2. Selection Flow Chart

Form April 2015 to May 2018, 211 patients with PCNs enrolled in this study. The selection strategy for the patients is described below. The first step was diagnosis according to clinical presentation and abdominal CT/MRI. The second step was whether it was feasible to perform the endoscopic ultrasonography-guided fine needle aspiration (EUS-FNA), and if it was, a 19G/22G needle was used (Cook Co., Boston, USA) along with a GF-UE260-AL5/GF-UM200 endoscope (Olympus Co., Tokyo, Japan) and an EU-ME2 endoscopic ultrasonography processor (Olympus Co., Tokyo, Japan). Needle puncture of the patient was used to obtain the fluid samples. The third step was the collection of cystic fluid for biochemical analysis, molecular diagnosis, and cytological examination. All patients with the following contraindications were excluded:

- pancreatic pseudocysts;
- severe acute pancreatitis;
- malignant tumours;
- severe cardiopulmonary circulatory system diseases;
- blood coagulation disorders.

Every patient provided informed consent. For those who did not undergo surgery, follow-up was conducted every 3 months, during which the EUS was reviewed to determine whether the cyst had become too large for EUS-FNA. Patients with concern of neoplastic growth underwent surgery but did not receive additional intervention.

### 2.3. Tumour Puncture and Extraction of Cystic Fluid

The patients were given intravenous anaesthesia by aspiration with oropharyngeal intubation. The most suitable puncture route was chosen so that important structures, such as the abdominal viscera and blood vessels, would be avoided in EUS-FNA. The lesions were punctured and connected to the negative pressure syringe, after which the cystic fluid was extracted at constant negative pressure. The extracellular cystic fluid was divided into two 1.0 mL cryopreservation tubes by a specialist, and a protease inhibitor was added to each tube at a ratio of 1:100. After the solution was mixed gently by vortexing, it was stored in a freezer at −80°C at the biological specimen bank. The frozen tubes were removed from storage during each experiment, which was performed after dissolution.

### 2.4. Lectin Microarray Analysis

Lectin microarrays were constructed and analysed by 37 commercial lectins from Vector Laboratories (Burlingame, CA, USA), Sigma-Aldrich (St. Louis, MO, USA), or Merck (Darmstadt, Germany). Cystic fluid protein samples were labelled with the fluorescent dye Cy3 (GE Healthcare; Buckinghamshire, UK) and were applied to the lectin microarrays. The slides were incubated in a humidity-controlled incubator at 50% humidity overnight to allow lectin immobilization. After incubation, the slides were blocked with blocking buffer (50 mM ethanolamine, 2% (w/v) BSA and 500 mM glycine in 50 mM sodium borate buffer, pH 8) for one hour and were then rinsed three times with PBST (0.05% Tween 20 in 0.1 M phosphate buffer containing 0.15 M NaCl, pH 7.4). This was followed by a final rinse in PBS (0.1 M phosphate buffer containing 0.15 M NaCl, pH 7.4). Before use, the slides were dried by centrifugation at 600 rpm for 5 minutes. After incubation, the slides were scanned with a GenePix 4000B confocal scanner (Axon Instruments; Union City, CA, USA).

### 2.5. Isolation and Purification of Glycoproteins

The cystic fluids from MCN group and SCN group were mixed in two main types and diluted in binding solution to a final concentration of 1 mg/mL and were mixed with 300 *μ*l of STL- or WGA-coupled magnetic particles, which were washed in binding solution. The mixtures were incubated for one hour at 25°C with shaking. Next, the magnetic particle conjugates were washed 5 times in washing solution to remove nonspecific proteins. Subsequently, the conjugates were incubated with elution buffer for one hour, and the glycoproteins enriched with STL or WGA were collected and quantitated by BCA assay.

### 2.6. Enzyme Cutting and Desalting

Before an appropriate amount of peptide sample was added, Lys-C hydrolytic enzyme was added in proportion to enzyme/protein at a ratio of 1:50; enzymolysis then occurred at 37°C for 6 hours. The sample was diluted with 50 mM NH4HCO3 solution so that the concentration of urea in the sample was lower than 1 M. Trypsin was added at an enzyme/protein ratio of 1:50, and the sample was incubated at 37°C in the enzyme solution overnight. Waters Oasis HLB solid-phase extraction (SPE) columns and an appropriate amount of 80% acetonitrile (ACN)/0.5% formic acid (FA) activated solid-phase extraction; this step was repeated twice. The FA balanced solid-phase extraction column step was also repeated twice. The enzymatic hydrolysis peptide sample flowed through the solid-phase extraction column, which was adsorbed on the filter. Moreover, the filtrate was collected, and passage through the column was repeated three times. Then, 0.5% FA was applied three times to clean the column. After washing the solid-phase extraction columns with an appropriate amount of 20% ACN/0.5% FA, 60% ACN/0.5% FA, and 80% ACN/0.5% FA, the three eluents that were collected were combined.

### 2.7. LC-MS/MS Analysis

LC-MS/MS analysis was performed on an EASY-nLC 1000 HPLC coupled with a Q Exactive™ mass spectrometer (Thermo Scientific, San Jose, CA, USA). Lyophilized peptide samples were resuspended in 0.1% FA and then separated on a reversed-phase C18 column in-house packed analytical column (75 *μ*m ID×20 cm, 3 *μ*m, ReproSil-Pur C18 AQ, Dr. Maisch GmbH, Germany) using mobile phase A (0.1% FA in water) and mobile phase B (0.1% FA in acetonitrile) with a gradient (5-8% B, 8 min; 8-22% B, 50 min; 22-32% B, 12 min; 32-95% B, 1 min; 95% B, 7 min) at a flow rate of 280 nL/min. The mass spectrometer was operated in positive ion mode and in the data-dependent acquisition mode. Full-scan MS spectra (from m/z 300 to 1600) were acquired in the Orbitrap at a high resolution of 70,000 (m/z 200) with an automatic gain control (AGC) of 3 × 10^6^ and a maximum injection time of 60 ms. The top 20 precursor ions were selected from each MS full scan with an isolation width of 2 m/z in the HCD collision cell at a normalized collision energy of 27%. Subsequently, MS/MS spectra were acquired in the Orbitrap with a resolution of 17,500 at m/z 200. The target value was 5 × 10^4^ with a maximum injection time of 80 milliseconds. Ions selected for MS/MS were dynamically excluded for a duration of 40 seconds. For nanoelectrospray ion source setting, the spray voltage was 2.0 kV, no sheath gas flow used, and the heated capillary temperature was 320°C. All raw data were viewed in Thermo Xcalibur version 2.2.

### 2.8. Data Analysis

The original data of microarrays need to be normalized by median normalization method for minimizing the possible systematic variation. The average background was subtracted, each sample was printed in triplicate, and data points with value outside of the average background ± standard deviation (SD) were removed from each data point. [24]. The normalized data of the MCN and SCN groups were compared with each other based on fold-change, according to the following criteria: p < 0.05 fold-change >1.45 or < 0.69; in pairs indicated upregulation or downregulation, respectively. The data were analysed by t-test using SPSS Version 22.0 (IBM, New York, USA).

Each enriched glycoprotein sample and the original mass spectrometry data were retrieved by the Proteome Discoverer™ Software. After the protein was identified, the intensity value was given, the protein intensity value was obtained for all three identifications, and the median value was extracted three times. The final protein intensity was expressed as the mean value of the three repetitions plus or minus the standard deviation value. The ratio of the normalized protein value in the MCN group to that in the SCN group was calculated to compare the relative changes in protein glycosylation. The fold-change > 1.21 as upregulated, and the fold-change <0.83 as downregulated, t-test and ratio criteria were selected as the final differential proteins. To better understand the WGA- and STL-binding glycoproteins in MCN and SCN, we applied the identified targets of interest to search tool for both UniProt database at https://www.uniprot.org and the Retrieval of Interacting proteins (STRING) 11.0 at http://string-db.org with a focus on the prediction with protein-protein interactions.

## 3. Result

### 3.1. Patient Characteristics

**Figure 2:**
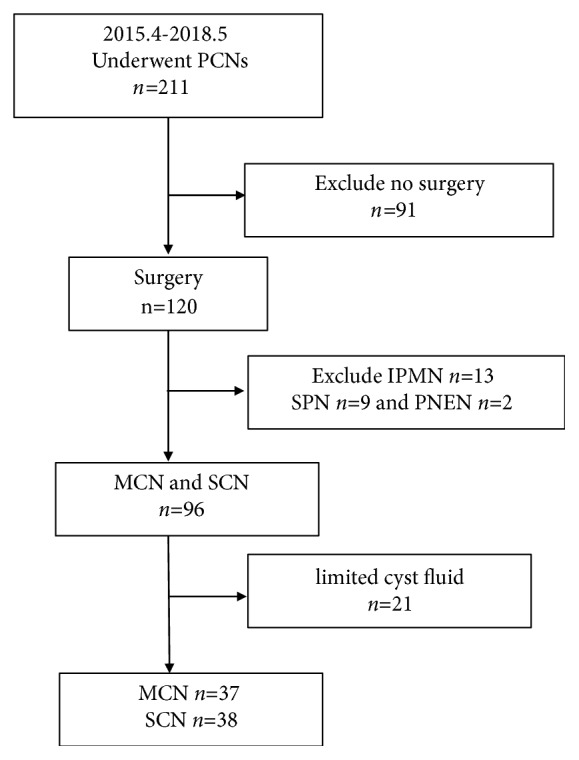
The flow chart of patients' selection.

About 211 PCNs were evaluated by EUS-FNA from April 2015 to May 2018. Among them, 88 were men and 123 were women. The average age was 57.36 and the standard deviation of the ages was 14.82. 120 patients (56.9%) were pathologically diagnosed through surgical resection, including 50 patients (42.5%) diagnosed with MCNs, 2 patients with MCN-low grade dysplasia, 2 patients with MCN-intermediate grade dysplasia, and 3 patients with MCN-associated invasive adenocarcinoma. 13 cases (10.8%) were diagnosed with IPMN. 46 patients (38.3%) were diagnosed with SCNs, with 9 patients (7.5%) diagnosed with SPN and 2 patients (1.7%) diagnosed with pancreatic neuroendocrine neoplasm (PNEN). A total of 96 cases were diagnosed as MCN or SCN. Sufficient amount of cystic fluid was harvested from 75 patients for glycopattern analysis. The flow chart of patient selection is illustrated in Figure 2.

### 3.2. Glycopattern Analysis of MCN and SCN

**Figure 3:**
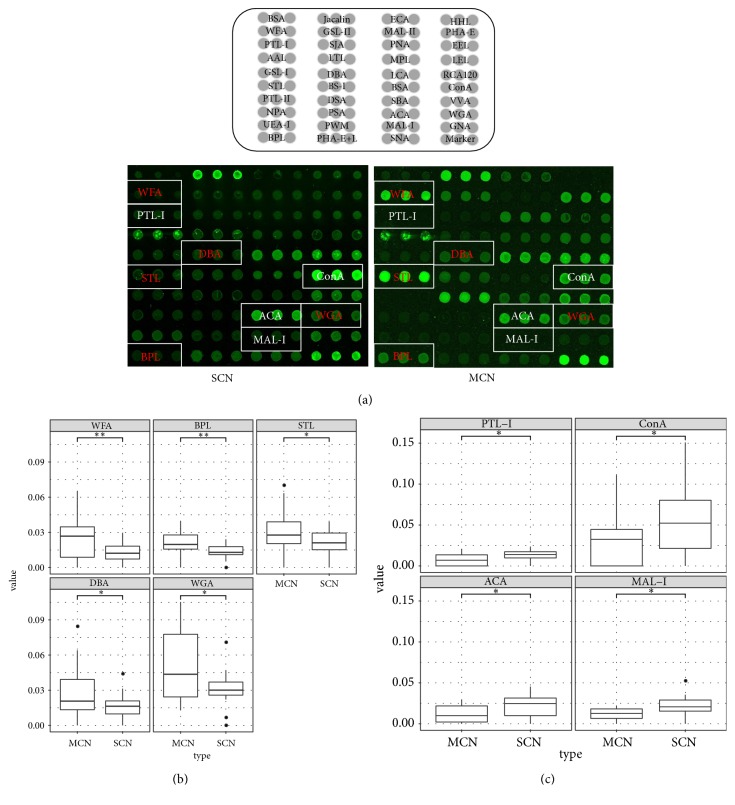
*Glycan-binding protein targeting SCN and MCN by lectin microarray*. (a) Lectin microarrays: representative MCN and SCN lectin microarrays: words in red indicate higher signal lectins in MCN compared with SCN, while words in white indicate lower signal lectins in MCN compared with SCN. (b) The recognition glycopattern of lectins was significantly higher in MCN samples than in SCN samples; box plots of lectins (R value > 1.45): WFA, DBA, STL, WGA, and BPL, *∗* p < 0.05, *∗∗*p<0.01. (c) The recognition glycopattern of lectins was significantly lower in the MCN samples than in the SCN samples; box plots of lectins (R value < 0.69): PTL-I, ConA, ACA, and MAL-I, *∗* p < 0.05, *∗∗*p<0.01.

The lectin microarray analysis, which included 37 lectins, 2 negative controls (BSA), and 1 positive control (Cy3-BSA), was performed to identify the glycopatterns of the two types of PCNs. The results revealed significant differences in the glycopatterns, as indicated by the white boxes (Figure 3(a)). Normalized relative signal intensities greater than 1.45 or lower than 0.69 were considered valid intensities. Based on intensity ratio of MCN to SCN, differential expressed lectins between two groups of patients were identified (Table 1). WFA, DBA, STL, and WGA were upregulated in MSN patients (Figure 3(b), MCN/SCN ratio > 1.45; p < 0.05), while PTL-1, Con A, and ACA were downregulated in MSN patients (Figure 3(c), MCN/SCN ratio < 0.69; p < 0.05).

### 3.3. Construction of Diagnostic Models Based on Glycopattern Abundances

**Figure 4:**
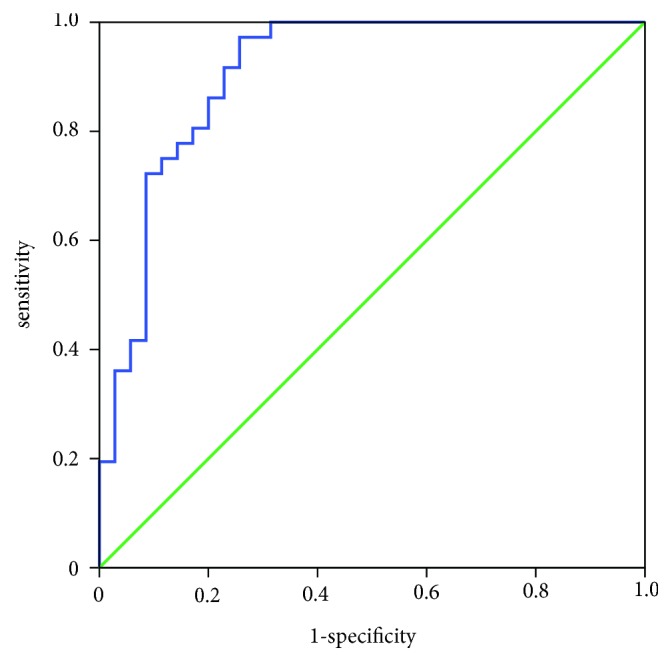
The diagnosis accuracy of the diagnostic models and selected lectins analysed by ROC analysis.

To test whether these lectins can be used to predict the types of patients, we built a logistic regression model using all lectins in the lectin microarray. The diagnostic accuracy of Model referred to four lectins: Erythrina Cristagalli Letin (ECA), Hippeastrum Hybrid Lectin (HHL), Triticum vulgaris Agglutinin (WGA), and Bauhinia Purpurea Lectin (BPL) were fed into logistic regression. The receiver operating characteristic (ROC) curve indicated that Model could distinguish MCN from SCN with high sensitivity and specificity (AUC: 0.907, sensitivity 0.971, and specificity 0.806) (Figure 4).

(1) Y=11+e−ECA×22.926+HHL×75.336+WGA×−58.112+BPL×−166.655+2.457

### 3.4. LC-MS/MS Analysis of MCN/SCN Glycoproteins by Differential Lectin Binding

**Figure 5:**
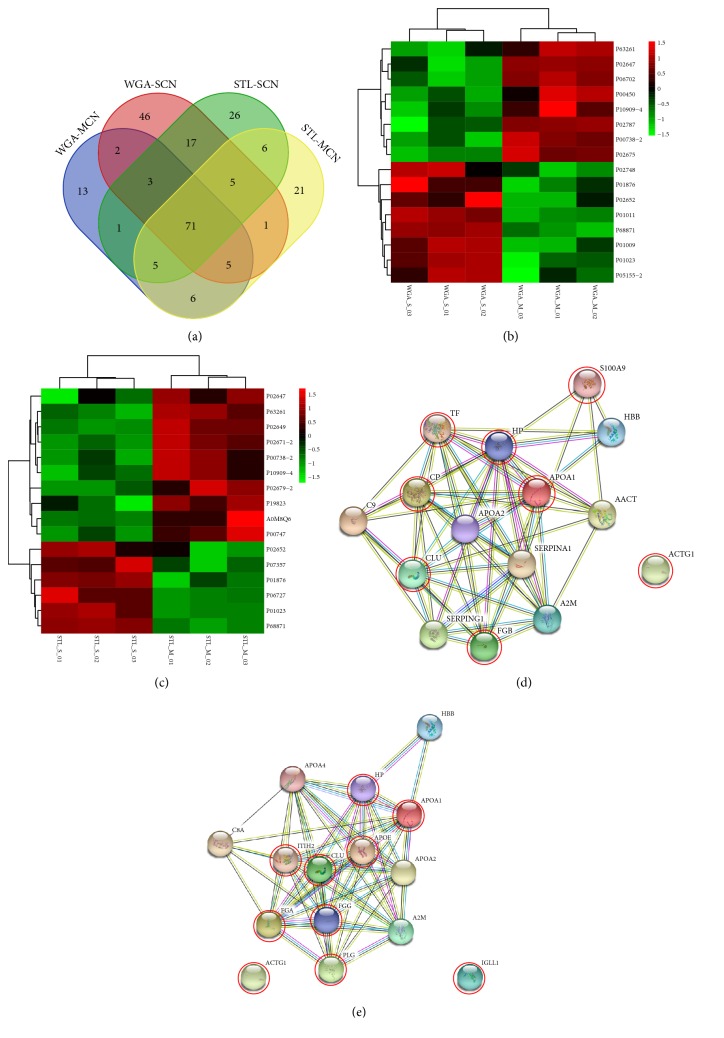
*Glycoproteins by differential lectin binding in MCN and SCN*. (a) Four-way Venn diagram: WGA- MCN, WGA-SCN, STL-MCN, and STL-SCN in a cross-statistic chart. (b) The Heat map of WGA-binding glycoproteins in MCN and SCN. The samples were listed in columns, and the proteins were listed in rows. The colour and intensity of each square indicated expression levels relative to the other data in the row. Red, high; green, low; black, medium. (c) The Heat map of STL-binding glycoproteins in MCN and SCN. The colour and intensity of each square indicated expression levels relative to the other data in the row. Red, high; green, low; black, medium. (d) String network interaction of APOA1, VTN, C5, and S100A9 genes significantly enriched in WGA-binding glycoproteomics. (e) String network interaction of APOE, ECM1, MUC5B, CP, KLKB1, and PLG genes significantly enriched in STL-binding glycoproteomics.

For the lectin affinity enrichment method, this study used the lectins STL and WGA to enrich glycosylated proteins and conducted LC-MS/MS analysis. In the three replications of each sample, the proteins identified in at least one of the replications were included in downstream analysis. 175 glycoproteins were enriched by WGA-coupled magnetic particles, of which 106 proteins and 150 proteins were identified in MSN group and SCN group, respectively. 167 glycoproteins were enriched by STL-coupled magnetic particles, of which 120 proteins and 134 proteins were in MSN group and SCN group, respectively (Table 2). According to the enriched lectins STL and WGA using peptide-spectrum matches (PSM) as the quantitative basis, a four-way WGA-MCN, WGA-SCN, STL-MCN, and STL-SCN Venn diagram shows the distribution of unique and shared proteins (Figure 5(a)).

The heat map in Figures 5(b) and 5(c) shows the expression of WGA- and STL-binding glycoproteins in MCN and SCN accordingly. In the three replications of each sample, these glycoproteins were based on the quantitative LC-MS/MS results, because the glycoproteins are mostly low-abundance proteins [25]. In this study, the label free quantification (LFQ) intensity was found generally in the order of e^8-9^. Sixteen differentially expressed proteins were detected in WGA-binding glycoproteins (p < 0.05), with eight upregulated proteins (MCN/SCN ratio > 1.21) and seven downregulated proteins (MCN/SCN ratio < 0.83) (Table 3). Sixteen differentially expressed proteins were identified in STL-binding glycoproteins (p < 0.05), and ten expressed upregulated glycoproteins and six downregulated glycoproteins (Table 4). Among these thirty-two different glycoproteins, the expressions of four glycoproteins (Apolipoprotein A-I, Actin gamma 1, Haptoglobin, and Clusterin) were increased in both lectin enrichment groups, and the expression of four glycoproteins (Immunoglobulin heavy constant alpha 1, Hemoglobin subunit beta, Apolipoprotein A-II, Alpha-2-macroglobulin) were decreased in both lectin enrichment groups.

### 3.5. STRING Aggregates and KEGG Pathways of DEGs

Using differentially expressed proteins identified, STRING aggregates and KEGG pathway were used to further annotate their functions. As one of the largest databases for protein-protein interaction, STRING aggregates the most available information derived from proteomic context, high throughput experiments, coexpression, and previous knowledge [26]. Using the STRING aggregates http://string-db.org, WGA-binding glycoproteins were mapped into an interaction network, and upregulated glycoproteins were in the red circle (Figure 5(d)), STL-binding proteins were mapped into an interaction network, and 10 increased expression were in the red circle (Figure 5(e)). Certain core genes have been reported to play important roles in pancreatic cystic neoplasms via KEGG pathway analysis. With the KEGG pathway enrichment analysis, the top three enriched pathways were complement and coagulation cascades, coagulation and fibrinolytic activation, and platelet activation (Table 5).

## 4. Discussion

Mammalian plasma membranes are known to contain 2% to 10% saccharides, most of which are oligosaccharides and glycoproteins. Linear or branched side chains of glycoproteins may contain two or more monosaccharide residues. At the end of the monosaccharide unit, there is often a negatively charged residue of N-acetylneuraminic acid and a siallic acid [27, 28]. Lectin is currently recognized as a monosaccharide or polysaccharide protein animals and plants and can be used to identify specific saccharide structure [29–31]. Different lectins have different binding affinity to glycan epitopes, which can be divided into mannose type, fucose type, sialic acid type/N-acetylglucosamine type, and Galactose type/N-acetylgalactose type [32, 33]. In order to improve the diagnostic accuracy of lectin microarray for MCN, we chose four lectins—Hippeastrum Hybrid Lectin (HHL) as the representative of mannose type, Triticum vulgaris Agglutinin (WGA) as the representative of sialic acid type/N-acetylglucosamine type, and Erythrina Cristagalli Letin (ECA) and Bauhinia Purpurea Lectin (BPL) as the representative of Galactose type/N-acetylgalactose type. The mathematical model was constructed to distinguish MCN from SCN with highest sensitivity and specificity (Figure 4).

In some researches, Shan Li has reported that oligosaccharide containing GalNAc and GlcNAc structural changes in Huh7 cells surface was associated with Epithelial Mesenchymal Transition (EMT) [34]. Gpc-1, an exosome-associated polysaccharide protein identified in the blood, has been shown to be significantly different among healthy individuals, pancreatic cancer patients, patients with early pancreatic cancer, and those with advanced pancreatic cancer [35]. Moreover, Gbormittah analysed pancreatic cystic tumours and found an increase in lectin WGA, which specifically recognized GlcNAc polymers and polyvalent sialic acid structure, accompanied by increasing level of MUC5AC and CA19-9 [36]. Due to the defect of lectin that one lectin can only enrich one class of glycoproteins, the method of multilectin binding is usually adopted to enrich glycoproteins. In this study, STL/WGA was selected to enrich glycoproteins to compare the differential lectin in MCN/SCN. The significance of the identified proteins was further shown by pathway analysis, protein-protein interactions, and chromosomal location investigations.

Seventy-five cyst fluids were collected by fine needle aspiration directly from the pancreatic cysts. In the cystic fluid of PCLs, Fibrinogen Alpha chain (FGA), fibrinogen beta chain (FGB), and fibrinogen gamma chain (FGG) were markedly elevated in MCN. They have been reported to elevate in prostate cancer, lung cancer, hepatocellular carcinoma, pancreatic cancer, infectious diseases, and myocardial infarction [37, 38]. Ghezzi reported that plasma fibrinogen level might be a potential index to predict prognosis, improving the early diagnosis of endometrial cancer and optimize the treatment schedule [39]. RT-PCR analysis showed that the expression level of FGG mRNA was significantly increased in adjacent tissues of liver cancer tissues, especially in liver cancer metastases [40].

Gene APOA1 is located on a well-known gene cluster at chromosome 11 and the accumulating evidence has shown that single nucleotide polymorphisms (SNPs) in the APOA1/A5/A4/C3 cluster are associated with high triglyceride (TG) levels [41]. Shavva found that the expression of endogenous APOA1 in human monocytes and macrophages was moderate and showed that proinflammatory cytokine tumour necrosis factor *α* (TNF*α*) increased APOA1 mRNA and stimulated APOA1 protein secretion by human monocytes and macrophages [42]. APOE was identified to be essential for TG-modulating, and three genes APOA1, APOA2, and APOE were reported to be associated with hypertriglyceridemia, which may increase the risk of acute pancreatitis [43].

S100 calcium binding protein A9 (S100A9) is a calcium- and zinc-binding protein which plays a prominent role in the regulation of inflammatory processes and immune response. It was reported to be dysregulated in many types of cancer, and it induced the overexpression of PD-1/PD-L1, which contributes to ineffective haematopoiesis in myelodysplastic syndromes [44]. Elevated serum S100A9 indicated poor prognosis in hepatocellular carcinoma after curative resection [45].

## 5. Conclusions

In summary, the study of alterations in glycoproteins associated with pancreatic cystic neoplasms can help in the search for tumour markers to improve cancer diagnosis. It may provide insights into the glycobiology mechanism underlying the progression of pancreatic cystic neoplasms and provide valuable information for the development of new therapies.

## Figures and Tables

**Table 1:** Differential Glycopattern Analysis of Glycoproteins in Cystic Fluid from MCN versus SCN Using Lectin Microarrays.

No.	Lectin	Binding structure	Normalized relative intensity		MCN/SCN Ratio
			MCN	SCN	
1	WFA	GalNAc*α*/*β*1−3/6Gal	0.026 ± 0.021	0.012 ± 0.009	2.13
2	DBA	GalNAc*α*-Ser/Thr(Tn) GalNAc*α*l-3Gal	0.028 ± 0.023	0.016 ± 0.011	1.76
3	STL	GlcNAc oligomer	0.030 ± 0.020	0.021 ± 0.011	1.47
4	WGA	Multivalent Sia and (GlcNAc)n	0.050 ± 0.029	0.031 ± 0.014	1.6
5	BPL	Gal*β*1-3GalNAc	0.021 ± 0.009	0.013 ± 0.006	1.56
6	PTL-I	*α*GalNAc and Gal	0.008 ± 0.007	0.013 ± 0.006	0.59
7	ConA	Branched and Terminal Man terminal GlcNAc	0.031 ± 0.029	0.066 ± 0.058	0.47
8	ACA	Gal*β*1-3GalNA*α*-Ser/Thr	0.012 ± 0.011	0.021 ± 0.013	0.57
9	MAL-I	Gal*β*-1,4GlcNAc	0.012 ± 0.007	0.021 ± 0.007	0.57

**Table 2:** LC-MS/MS analysis of Glycoproteins in MCN versus SCN.

WGA-SCN	WGA-MCN	WGA	STL-SCN	STL-MCN	STL
150	106	175	134	120	167

**Table 3:** The protein enrichment by WGA in MCN versus SCN.

Protein name	Gene name	Majority protein IDs	Normalized relative intensity		MCN/SCN Ratio
			MCN	SCN	
(A) Increased expression					
Ceruloplasmin	CP	P00450	3.58 ± 0.45	2.38 ± 0.15	1.50
Haptoglobin	HP	P00738-2	21.43 ± 1.85	11.86 ± 1.31	1.81
Apolipoprotein A-I	APOA1	P02647	203.23 ± 0.80	163.91 ± 9.36	1.24
Fibrinogen beta chain	FGB	P02675	55.94 ± 5.12	32.06 ± 1.62	1.74
Serotransferrin	TF	P02787	47.99 ± 0.83	28.92 ± 4.17	1.66
Protein S100-A9	S100A9	P06702	4.82 ± 0.38	2.08 ± 0.28	2.32
Clusterin	CLU	P10909-4	9.19 ± 0.84	6.99 ± 0.42	1.32
Actin gamma 1	ACTG1	P63261	3.69 ± 0.30	2.59 ± 0.27	1.43
(B) Decreased expression					
Serpin peptidase inhibitor clade A member 1	SERPINA1	P01009	41.05 ± 2.37	53.74 ± 2.02	0.76
Alpha-1-antichymotrypsin	AACT	P01011	3.88 ± 0.22	7.91 ± 0.52	0.49
Alpha-2-macroglobulin	A2M	P01023	15.58 ± 2.81	33.14 ± 3.32	0.47
Immunoglobulin heavy constant alpha 1	IGHA1	P01876	27.16 ± 3.34	43.11 ± 7.02	0.63
Apolipoprotein A-II	APOA2	P02652	44.02 ± 1.52	49.89 ± 2.11	0.88
Complement component 9	C9	P02748	3.21 ± 0.14	3.89 ± 0.24	0.83
Plasma protease C1 inhibitor	SERPING1	P05155	9.05 ± 3.04	26.13 ± 5.88	0.35
Hemoglobin subunit beta	HBB	P68871	86.97 ± 13.09	320.97 ± 14.96	0.27

**Table 4:** The protein enrichment by STL in MCN versus SCN.

Protein name	Gene name	Majority protein IDs	Normalized relative intensity		MCN/SCN Ratio
			MCN	SCN	
(A) Increased expression					
Ig lambda-7 chain C region	IGLL1	A0M8Q6	12.35 ± 10.23	1.38 ± 0.08	8.95
Haptoglobin	HP	P00738	15.42 ± 1.67	10.13 ± 0.88	1.52
Plasminogen	PLG	P00747	14.19 ± 1.29	10.48 ± 0.50	1.35
Apolipoprotein A-I	APOA1	P02647	195.25 ± 8.29	160.44 ± 14.22	1.22
Apolipoprotein E	APOE	P02649	48.10 ± 2.26	34.68 ± 0.53	1.39
Fibrinogen Alpha A	FGA	P02671	44.56 ± 3.31	29.25 ± 1.01	1.52
Fibrinogen gamma chain	FGG	P02679	35.71 ± 4.92	21.61 ± 1.32	1.65
Clusterin	CLU	P10909	8.70 ± 1.07	5.36 ± 0.50	1.62
Inter-alpha-trypsin inhibitor heavy chain H2	ITIH2	P19823	10.27 ± 0.70	6.62 ± 0.96	1.55
Actin, gamma 1	ACTG1	P63261	4.21 ± 0.32	2.34 ± 0.18	1.8
(B) Decreased expression					
Alpha-2-macroglobulin	A2M	P01023	13.27 ± 0.43	35.82 ± 4.42	0.37
Ig alpha-1 chain C region	IGHA1	P01876	18.50 ± 1.83	29.41 ± 0.56	0.63
Apolipoprotein A-II	APOA2	P02652	39.36 ± 7.77	62.11 ± 7.96	0.63
Apolipoprotein A-IV	APOA4	P06727	2.45 ± 0.09	5.36 ± 1.04	0.46
Complement component C8 alpha chain	C8A	P07357	2.17 ± 0.12	2.73 ± 0.16	0.8
Hemoglobin subunit beta	HBB	P68871	97.91 ± 6.10	308.30 ± 10.39	0.32

**Table 5:** KEGG pathway analysis of the DEGs in MCN versus SCN.

Pathway name	Genes number	Genes ID
hsa 04610:Complement and coagulation cascades	10	CLU, A2M, FGA, FGB, FGG, PLG, SERPINA1, SERPING1, C8A, C9
hsa 04611:Platelet activation	4	FGA, FGB, FGG, ACTG1
hsa 04979:Cholesterol metabolism	3	APOA1, APOE, APOA2, APOA4

## Data Availability

The data used to support the findings of this study are included within the article.
